# A comparison of the responsible drinking dimensions among underage and legal drinkers: examining differences in beliefs, motives, self-efficacy, barriers and intentions

**DOI:** 10.1186/1747-597X-9-5

**Published:** 2014-01-22

**Authors:** Adam E Barry, Michael L Stellefson, Conrad L Woolsey

**Affiliations:** 1 Department of Health Education & Behavior, University of Florida, P.O. Box 118210, Gainesville, FL 32611, USA; 2 Nutrition and Human Performance, Logan University, Chesterfield, MO 63017, USA

**Keywords:** Alcohol, Underage drinking, Responsible drinking, College, Amethyst initiative, Minimum legal drinking age

## Abstract

**Background:**

To date, scholarly discourse over the Amethyst Initiative has primarily debated the relative effectiveness of the 21 year-old Minimum Legal Drinking Age (MLDA). Unfortunately, this discourse has failed to account for the Amethyst Initiative’s central tenet/mission: *facilitating responsible drinking among college students*. This investigation seeks to help fill this gap by quantitatively determining whether a random sample of underage (*n* = 158) and legal (*n* = 298) drinkers differed with regard to their alcohol-related behaviors, responsible drinking behaviors, and responsible drinking beliefs.

**Findings:**

Compared to legal drinkers, underage drinkers reported: (a) significantly less confidence to perform responsible drinking behaviors during their next drinking episode [t(446) = -2.97, *p* < .003; *d* = -0.297], (b) significantly more perceived barriers to responsible drinking [t(388) = 3.44, *p* < .001; *d* = .368], and (c) significantly lower behavioral intentions to perform responsible drinking behaviors the next time they consumed alcohol [t(437) = -3.45, *p* < .001; *d* = -0.350]. Each of these differences remained statistically significant, even after controlling for sex and race, in three separate multiple linear regression models.

**Conclusion:**

While college students both above and below the 21 year-old MLDA have similar beliefs regarding what constitutes responsible drinking, students below the current MLDA have less intention to drink responsibly regardless of their behavioral beliefs and/or motives. College/university administrators should consider the negative repercussions that are possible if underage students who are less confident in their ability to drink responsibly are given the legal right to drink on campus.

## 

Debate over the United States’ minimum legal drinking age (MLDA) has recently erupted [1-4]. Despite epidemiological data linking the current MLDA of 21 with reduced alcohol-related mortality, morbidity, and traffic crashes [5-8], a substantial number of university chancellors and presidents have signed a public statement seeking informed, dispassionate discourse over the 21 year-old MLDA [9]. Referred to as the *Amethyst Initiative*, this proposal supports a series of educational and policy level efforts to enable 18–20 year old adults to purchase, possess, and consume alcoholic beverages at their own discretion.

To date, discourse over the Amethyst Initiative has primarily revolved around the relative effectiveness of the 21 year-old MLDA. This focus seems counterproductive for several reasons. First, it is difficult to dispute the efficacy of the MLDA [8]. Second, focusing on the policy’s effectiveness fails to spotlight the Amethyst Initiative’s core mission: *facilitating responsible drinking among college students*. There is a dearth of literature investigating differences in how underage and legal drinkers practice and/or conceptualize responsible drinking behavior(s) specifically, yet studies have explored other aspects of responsible drinking among college students [10-12]. Consequently, this investigation seeks to quantitatively determine whether underage and legal drinkers differ on their alcohol-related behaviors, and responsible drinking beliefs, motives, self-efficacy, barriers and intentions.

## Methods

A random sample of college students attending a large, Southwestern, four-year public university were asked to voluntarily complete a web-based survey in an uncontrolled setting (e.g., on a respondent’s home computer). Respondents were selected from a master list obtained from the university registrar that contained contact information (name and e-mail) for all enrolled undergraduate students. The survey took approximately 20 minutes to complete. Respondents were made aware that they would be entered into a lottery drawing for an MP3 player to incentivize participation. All procedures were vetted and approved by the university’s Institutional Review Board (protocol #2006-0428).

### Measures

#### ***Alcohol-related behaviors***

*Most Recent Drinking Episode* assessed how many alcoholic drinks were consumed the last time in a social setting with alcohol. Respondents typed in the number of drinks they consumed during this event. *Binge Drinking* assessed how many times respondents consumed five or more alcoholic drinks at a sitting within the past two weeks. Nine possible response options ranged from ‘0’ to ‘9 or above’.

#### ***Responsible drinking***

The *Characteristics of Responsible Drinking Survey* (CHORDS) [13] assessed several responsible drinking dimensions, including one’s behavioral beliefs, motivations, self-efficacy, barriers, and behavioral intentions regarding the responsible consumption of alcohol (see Barry & Goodson [13] for more detailed psychometric information and item wording).

*Behavioral Beliefs* (α = .82) were assessed using 8 items that measured behaviors ranging from drinking and driving, to maintaining a blood alcohol concentration (BAC) below the legal limit (0.08%). Response options included *never*: not important to do when drinking any alcohol (0); *seldom*: would be nice to do but not necessary (1); *some of the time*: only when it is possible (2); *most of the time*: should try to do this (3); or *always*: must do this every time he/she drinks any alcohol, no matter what (4).

*Motivations* (α = .86) were determined using 21 items assessing the extent to which various intrapersonal (e.g., religious convictions), interpersonal (e.g., desire not to upset significant others or parents), and other contextual factors (e.g., having to drive home, work- and school-related obligations) facilitate responsible drinking. Response options included (0) never, (1) seldom, (2) some of the time, (3) most of the time, or (4) always.

The 8-item *Self-efficacy* scale (α = .87) assessed perceived confidence in performing each of the actions outlined in the *Behavioral Beliefs* scale described above. Self-efficacy was measured using a scale from 0% (having no confidence) to 100% (extremely confident), with respondents given the option to select their level of confidence for each alcohol behavior in 10% increments (i.e., (1) 10% confident, (2) 20% confident, (3) 30% confident, etc.).

The *Barriers* scale (α = .91) encompasses 16 items which examine circumstances (e.g., felt depressed or stressed) and contextual factors (e.g., recently broken-up with a significant other, an attractive person wanted to buy you a drink) that could impede someone from drinking responsibly. Respondents indicated whether each item would be an obstacle to drinking responsibly (0) never, (1) seldom, (2) some of the time, (3) most of the time, or (4) always.

The 8-item *Behavioral Intentions* scale (α = .84) assessed the likelihood of performing the actions outlined in the *Behavioral Belief* scale. Specifically, respondents indicated whether they were (0) not likely at all, (1) seldom likely, (2) somewhat likely, (3) likely, or (4) extremely likely, to perform responsible drinking behaviors, the next time they chose to drink.

#### ***Handling missing data***

Only a small percentage of respondents had missing data on any of the subscales (2.0% of *Behavioral Beliefs*, 5.0% of *Motivations*, 1.3% of *Self-Efficacy*, 6.8% of *Barriers*, and 3.5% of *Behavioral Intentions*). Respondents with incomplete data did not differ from those with fully completed surveys with regards to: sex [t(457) = .194, *p* = .846], age [t(454) = -.797, *p* = .426], Greek (fraternity/sorority) status [t(453) = -.807, *p* = .420], full-time student status [t(456) = -.445, *p* = .656], binge drinking status [t(455) = .172, *p* = .864], or the number of days in which alcohol was consumed in the past 30 days [t(457) = .900, *p* = .369]. Consequently, incomplete surveys were retained for analysis.

#### ***Data analysis***

Among underage and legal drinkers, independent sample t-tests were performed to compare mean scores on the continuous alcohol-related behaviors and 5 subscales of the CHORDS. Effect sizes (Cohen’s *d*) were computed for all statistically significant mean differences. All between-group differences found to be statistically significant were further explored in multiple linear regression models, controlling for sex and race/ethnicity.

## Results

459 self-identified underage (*n* = 158; 35%) and legal (*n* = 298; 65%) drinkers completed the web-based survey. Participants were primarily full-time students (93%) who were Caucasian (79.3%) and female (54.5%), with a mean age of 22 years (SD = 5.47 years). Approximately 11% of respondents were members of a fraternity or sorority, and the majority (78%) resided in off-campus housing or an on-campus residence hall (18%). There was an equal representation (~ 18% per each year in school) across all student classifications (e.g., freshmen, sophomore). Both gender and ethnic distributions of those surveyed were comparable to the institutional population from which the sample was drawn (47% female, 73% Caucasian, 11% Hispanic, 4% Asian, and 3% Black).

Table 1 reports mean differences in alcohol consumption and responsible drinking beliefs and behaviors among underage and legal drinkers. Underage drinkers (*M* = 4.44, SD = 2.95) and legal drinkers (*M* = 4.10, SD = 3.29) consumed similar amounts of alcohol the last time they were in a social situation [t(450) = 1.09, *p* = .277]. Likewise, underage drinkers (*M* = 1.44, SD = 1.73) and legal drinkers (*M* = 1.26, SD = 1.88) reported comparable rates of binge drinking [t(452) = 1.02, *p* = .310]. Compared to legal drinkers (*M* = 7.18, SD = 2.06), however, underage drinkers (*M* = 6.58, SD = 1.96) reported significantly less self-efficacy to perform responsible drinking behaviors during their next drinking episode. The magnitude of difference (i.e., effect size) in self-efficacy (mean difference = -.60; 95% CI: -.99 to -.20) was relatively small, however (Cohen’s *d* = -0.297). Underage drinkers (*M* = 1.57, SD = .79) also reported significantly more perceived barriers to responsible drinking compared to their of-age counterparts (*M* = 1.27, SD = .80). The magnitude of difference in barrier means between groups (mean difference = .29, 95% CI: .13 to .46) was medium (Cohen’s *d* = .368). Moreover, compared to legal drinkers (*M* = 2.57, SD = .83), underage drinkers (*M* = 2.29, SD = .78) also reported significantly lower behavioral intentions to perform responsible drinking behaviors the next time they consumed alcohol (mean difference = -.28, 95% CI: -.44 to -.12, Cohen’s *d* = -0.350). No statistically significant differences were reported between groups on responsible drinking behavioral beliefs or motivations.

**Table 1:** Differences in alcohol consumption and responsible drinking beliefs and behaviors among underage and legal drinkers

**Constructs**	**Underage drinker**	**Legal drinker**	***t***	***df***	***p*-value**	**Cohen’s *d***
***Alcohol-Related Behaviors***						
Most Recent Drinking Episode	4.44	4.10	1.09	450	.277	**--**
	(2.95)	(3.29)				
Binge Drinking	1.44	1.26	1.02	452	.310	**--**
	(1.73)	(1.88)				
***Responsible Drinking Beliefs & Behaviors***						
Behavioral Beliefs	2.68	2.75	-1.10	445	.272	**--**
	(.68)	(.68)				
Motivations	2.06	2.07	-.10	411	.924	**--**
	(.63)	(.62)				
Self-Efficacy	6.58	7.18	-2.97	446	.003	-.297
	(1.96)	(2.06)				
Barriers	1.57	1.27	3.44	388	.001	.368
	(.79)	(.80)				
Behavioral Intentions	2.29	2.57	-3.45	437	.001	-0.350
	(.78)	(.83)				

Table 2 presents findings from three separate multiple linear regression models, each of which examined the relationship between legal drinking status and the three statistically significant responsible drinking dimensions in Table 1 (i.e., self-efficacy, barriers, intentions). In all three models, legal drinking status predicted each responsible drinking dimension to a statistically significant degree, above and beyond sex and race/ethnicity. In regards to barriers to responsible drinking, only legal drinking status (β = -.17) was statistically significant, such that the number of factors inhibiting responsible drinking decreased with a one unit increase in the independent variable (i.e., moving from underage to over the MLDA). For both responsible drinking self-efficacy (β = .15) and behavioral intentions (β = .16), going from underage to over the MLDA resulted in greater confidence and increased intention to demonstrate responsible drinking behavioral beliefs.

**Table 2:** **Multiple linear regression analyses predicting responsible drinking self-efficacy, barriers & intentions in underage and legal drinkers (*n*** **= 456)**

**Variable**		***B***	***SE B***	***β***	***t***	***p***
**Self-Efficacy to Drink Responsibly**						
Constant		7.013	0.347		20.238	0.001
Male		-0.673	0.191	-0.164***	-3.524	0.001
White		-0.224	0.314	-0.044	-0.711	0.477
Hispanic		0.319	0.427	0.046	0.746	0.456
21 or older		0.626	0.199	0.146**	3.140	0.002
*R*^*2*^	.044					
*F*	6.143***					
*df*	4, 441					
**Barriers Inhibiting Responsible Drinking**						
Constant		1.554	0.159		9.749	0.001
Male		0.025	0.082	0.016	0.309	0.757
White		0.017	0.147	0.008	0.118	0.906
Hispanic		-0.087	0.189	-0.033	-0.461	0.645
21 or older		-0.296	0.086	-0.174***	-3.450	0.001
*R*^*2*^	.021					
*F*	3.078*					
*df*	4, 383					
**Behavioral Intentions to Drink Responsible**						
Constant		2.675	0.141		18.922	0.001
Male		-0.409	0.076	-0.248***	-5.365	0.001
White		-0.221	0.128	-0.106	-1.727	0.085
Hispanic		-0.181	0.175	-0.064	-1.038	0.300
21 or older		0.278	0.079	0.161***	3.503	0.001
*R*^*2*^	.081					
*F*	10.660***					
*df*	4, 432					

*p < .05.

**p < .010.

***p < .001.

## Discussion

Despite a lack of empirical evidence suggesting that lowering the MLDA will curb binge drinking among college students, [14] the *Amethyst Initiative* continues to attract supporters and attention [15]. Underage drinkers in our sample reported a significantly greater number of factors that would inhibit their ability to drink responsibly as compared to students over the MLDA. Underage students also reported significantly less self-efficacy and lower intentions to engage in responsible drinking behaviors the next time they consumed alcohol. While college students both above and below the MLDA have similar beliefs regarding what constitutes responsible drinking, those below the current MLDA have less intention to drink responsibly regardless of their behavioral beliefs and/or motives. While we cannot make definitive claims about potential changes in drinking behaviors, or responsible drinking beliefs and behaviors, of 18–20 year olds if the MLDA were lowered, our findings do illustrate noteworthy differences in responsible drinking dimensions among illegal and legal college student drinkers. Other research suggests that college students who endorse a personal responsibility to obey the current MLDA of 21 are in the minority [16], and heavier and riskier drinkers are more likely to contend the MLDA should be lower than their lighter drinking peers [17]. Future research therefore, should further explore potential causal effects of the proposed MLDA reduction among college students.

## Conclusion

Current evidence suggests that age-based restrictions on access to alcohol have substantial impact on alcohol consumption, such that the MLDA clearly reduces alcohol consumption and its associated harms [18]. Granting increased access to alcohol by lowering the MLDA could lead to increased rates of drinking and subsequent alcohol-related consequences [19]. For example, after New Zealand lowered its MLDA from 20 to 18, there were substantial increases in alcohol-related hospitalizations [20]. Recent system models simulating lowered MLDA changes also suggest “pessimistic” outcomes resulting from the *Amethyst Initiative*, including an increased social availability of alcohol (campus wetness) that will likely overshadow any anticipated benefits stemming from allowing those 18 and older to consume alcohol legally [21]. Therefore, university chancellors and presidents should strongly consider Fitzpatrick et al.’s [21] warning that, “lowering the current MLDA represents an enormous social experiment with potentially major consequences” (p2).

## Competing interests

The authors declare that they have no competing interests.

## Authors’ contributions

AEB conceptualized the manuscript and prepared the first draft of the manuscript. MLS and CW contributed to the introduction and review of literature. MLS consulted on the statistical analysis. AEB, MLS and CW contributed to the conceptual development of the discussion and conclusion section. Both MLS and CW reviewed the study design, statistical approaches, and each successive draft of the manuscript. All authors contributed to the overall construction of this manuscript, read, edited, and approved the final draft of the manuscript. AEB takes responsibility for the paper as a whole.

## Authors’ information

The findings reported herein build upon AEB’s previous work, which examined how college students interpret and practice “responsible drinking”. As readers will note, the responsible drinking dimensions reported herein, and elsewhere, echo those of previous investigations focusing on protective behavioral strategies (PBS) drinkers employ to stay safe while drinking. Thus, this manuscript contributes to both the scant literature base focused on responsible drinking, as well as the larger burgeoning literature base focused on harm reduction strategies.
